# Protocol for long-term *ex vivo* cultivation and imaging of *Drosophila* imaginal discs

**DOI:** 10.1016/j.xpro.2025.103876

**Published:** 2025-06-09

**Authors:** Liyuan Sui, Christian Dahmann

**Affiliations:** 1 School of Science, Technische Universität Dresden, 01062 Dresden, Germany; 2 Cluster of Excellence Physics of Life, Technische Universität Dresden, 01062 Dresden, Germany

**Keywords:** Cell Biology, Developmental biology, Model Organisms

## Abstract

*Drosophila* wing and eye-antennal imaginal discs are excellent models for studying tissue development and cell behavior. Here, we present a protocol for long-term cultivation and imaging of wing and eye-antennal imaginal discs. We describe the mounting, dissection, and *ex vivo* cultivation of imaginal discs. We further detail 4D time-lapse image acquisition with minimized phototoxicity and image restoration using a machine-learning-based algorithm. This protocol is suitable for studying dynamic cell behaviors with single-cell resolution during normal and perturbed development.

For complete details on the use and execution of this protocol, please refer to Sui and Dahmann.^1^

## Highlights

• Steps for dissection, mounting, and *ex vivo* cultivation of imaginal discs
• Instructions for live imaging of *ex vivo* cultured imaginal discs
• Guidance for denoising and restoration of axial resolution of images

## Abstract (editor highlights)

Publisher’s note: Undertaking any experimental protocol requires adherence to local institutional guidelines for laboratory safety and ethics.

## Graphical abstract

**Figure:**
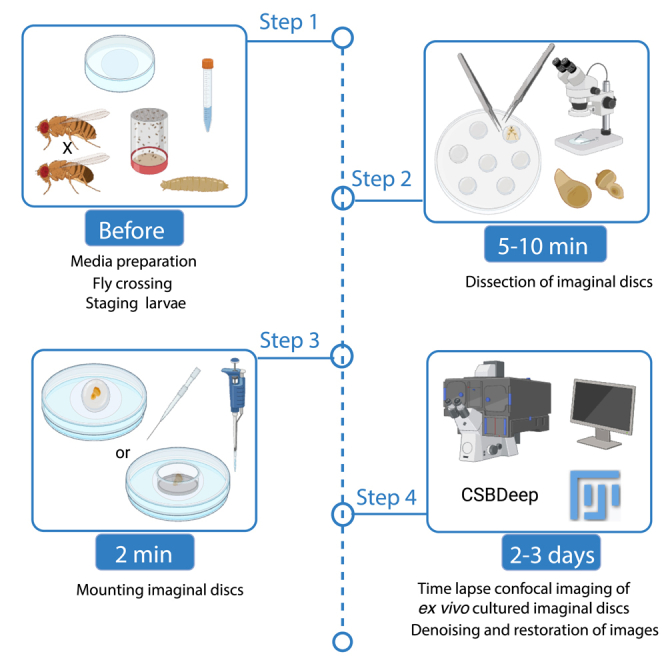

## Before you begin

### Preparing Grace’s medium

**Timing: 1 h**

1. Prepare Grace’s medium.
  a. Add one bottle of Grace’s medium powder and 0.35 g NaHCO_3_ into 900 ml sterile distilled water and stir to dissolve.
  b. Adjust pH with 1 M NaOH to 6.5 at room temperature.
  c. Add sterile water to 1 L.
  d. Filter media through a 0.22 μm filter (Millipore).
  e. Dispense the solution into 4.5 ml aliquots and keep them at 4°C. The aliquoted media can be stored at 4°C for 4–5 months.

### Preparing culture medium

**Timing: 10 min**

2. Prepare culture medium on the day of imaging.

To aliquoted Grace’s medium (4.5 ml) add 250 μL Fetal bovine serum, 200 μL sterile distilled water, 50 μL Penicillin streptomycin and 5 μL 0.01 mg/ml 20-Hydroxyecdysone. The culture medium is optimized based on Dye et al., 2017.^2^ Troubleshooting 1–3.

***Note:*** Addition of 200 μL sterile distilled water to Grace’s medium balances the osmotic pressure of the culture medium with the intracellular osmotic pressure of the tissue.

**CRITICAL:** Addition of 5 μL 0.01 mg/ml 20-Hydroxyecdysone into Grace's medium (4.5 ml) is optimized for cultivation of imaginal discs from early- and mid-third instar larvae.

### Preparing poly-D-lysine-coated dishes

**Timing: 2 days**

3. Prepare Poly-D-Lysine coated dishes.
  a. Add 600 μL 0.1 mg/ml of Poly-D-Lysine in the middle of a glass bottom microwell dish.
  b. Place dish overnight at 4°C.
  c. On the next day, remove surplus Poly-D-Lysine liquid from the dish.
  d. Dry the dish under a hood for 1 h.

***Note:*** Remove as much of the surplus Poly-D-Lysine solution from the dish as possible before drying the dish in the hood. This will help prevent the formation of crystals on the bottom of the dish after drying. Troubleshooting 4.

### Preparing flies

**Timing: 2 weeks**

To observe the behavior of single cells, generate clones of cells expressing appropriate fluorescent markers using the FRT/Flp^3^ and Gal4/UAS^4^ systems. In addition, all cells of the imaginal discs can be labeled with a different fluorescent marker (e.g. Indy-GFP, which labels cell membranes).

4. Drosophila husbandry.
  a. To generate clones in which cell membranes are marked, use stocks of genotype *hsp-flp; Act5C>CD2>Gal4* and *UAS-CD8-GFP.*
  b. To generate clones in which the cytoplasm is marked, use stocks of genotype *hsp-flp; Act5C>CD2>Gal4* and *UAS-RFP.*
  c. To generate clones in which F-actin is marked, use stocks of genotype *hsp-flp; Act5C>CD2>Gal4* and *UAS-Lifeact-GFP.*
  d. To generate clones in which nuclei are marked, use stocks of genotype *hsp-flp; Act5C>CD2>Gal4* and *UAS-His-RFP.*
  e. To additionally label cell outlines, use stock of genotype *Indy-GFP.*
  f. To label adherens junctions, use stock of genotype *DE-cad::mTomato.*

### Preparing and staging larvae

**Timing: 2 weeks**

5. Crossing flies.
  a. Cross virgin female flies of genotype *hsp-flp; Act5C>CD2>Gal4* with male flies carrying a gene encoding a fluorescent marker under control of *UAS* (e.g. *UAS-CD8-GFP*).
  b. Raise flies for 5–7 days in standard food vials.^5^ Change vials every 2–3 days.

***Note:*** The adult flies should be 5 to 7 days old before collecting eggs. Flies that are either too old or are too young will not generate sufficient eggs in a short time.

6. Stage larvae.
  a. Transfer 30–50 adult flies with 70% females and 30% males to a fly cage containing apple juice agar plate.^6^
  b. Raise flies in the fly cage overnight at 25°C.
  c. Discard the apple juice agar plate of the fly cage.
  d. Renew the apple juice agar plate containing yeast every two hours, for 1 day.
  e. Collect the apple juice agar plate.
  f. Remove the yeast.
    ***Note:*** Removing the yeast prevents the fermentation of the fly food.
  g. Add fresh fly food in the center of the apple juice agar plate.
  h. Place a lid onto the apple juice agar plate and incubate at 25°C for 1 day.
  i. Transfer larvae to a food vial.
7. Generate clones of cells expressing the fluorescent marker
  a. Subject 72 h - 84 h after egg lay (AEL) larvae to a 10 min heat-shock at 37°C in a water bath.
    ***Note:*** Place the food vial sufficiently deep into the water bath that the entire region of the food vial containing the larvae is submersed under water, while avoiding that water enters the food vial.
  b. Incubate larvae at 25°C.
8. Dissect larvae ∼12 - 15 h after the heat shock.

### Installation of software

**Timing: 1 h**

9. Install CSBDeep plugin from the Fiji update site.
10. Install a Graphic card, Python, TensorFlow and jupyter notebook on your computer
  https://github.com/CSBDeep/CSBDeep/tree/main/.github.

## Key resources table

**Table:** 

REAGENT or RESOURCE	SOURCE	IDENTIFIER
**Chemicals, peptides, and recombinant proteins**		

Grace’s medium powder	Sigma	G9771
Fetal bovine serum	Gibco	16000-036
Penicillin streptomycin	Sigma	P4333
20-Hydroxyecdysone	Sigma	H5142
0.1 mg/ml poly-D-lysine	Gibco	A3890401
NaHCO_3_	Sigma	S2127

**Experimental models: Organisms/strains**		

*D. melanogaster: DE-Cad::mTomato*	Huang et al.^7^	N/A
*D. melanogaster: UAS-CD8-GFP*	Bloomington Drosophila Stock Center	5137
*D. melanogaster: UAS-Lifeact-GFP*	Bloomington Drosophila Stock Center	35544
*D. melanogaster: Indy-GFP*	Quinones-Coello et al.^8^	N/A
*D. melanogaster*: *hsp-flp*;; *Act5C>CD2>Gal4*	Pignoni and Zipursky^9^	N/A
*D. melanogaster: UAS-RFP*	Bloomington Drosophila Stock Center	31417
*D. melanogaster: UAS-His-RFP*	Bloomington Drosophila Stock Center	76361

**Software and algorithms**		

Fiji	Schindelin et al.^10^	RRID: SCR_002285

**Other**		

35 mm Petri dish	MatTek	No.P35G-1.5-20-C
60 × 15 mm small Petri plates	Edvotek	Ca#633
Cell culture inserts	Millicell	PI8P01250
0.22 μm filter	Millipore	SLGL0250S
Forceps, Dumont no. 5	Sigma-Aldrich	F6521
Dissecting needle	Fisher Scientific	13-820-024

## Step-by-step method details

### Live imaging of *ex vivo* cultured imaginal discs

**Timing: 1–24 h**

The following steps detail the dissection, mounting and *ex vivo* cultivation of wing and eye-antennal discs for live imaging.

1. Wash larvae.
  a. Float larvae out of the food by distilled water. Transfer larvae using forceps into 60 mm sterile petri dishes containing 10 ml PBS.
  b. Clean larvae by transferring larvae into new petri dishes containing 10 ml PBS; repeat 3 times.
  c. Prepare 15 droplets of culture medium (30 μL per droplet) in the lid of a sterile petri dish. Wash one larva one more time in one of the droplets of culture medium.

***Note:*** It is not necessary to wash the larvae in ethanol, as ethanol can easily adhere to the larvaes’ cuticle and contaminate the culture medium. Do not keep larvae in PBS for more than 2 hours.

**Figure 1:**
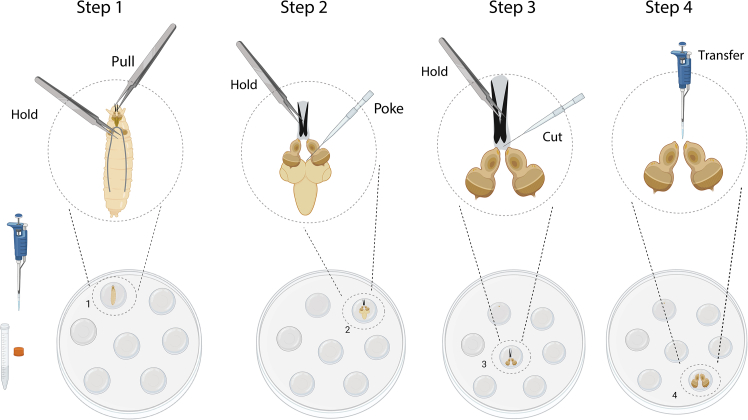
Dissection of eye-antennal discs for *ex vivo* culture Step-by-step procedure for isolating eye-antennal discs from third instar larvae. Step 1: Use forceps to gently pull out the mouth hooks from the larvae to expose the eye-antennal discs and the associated larval brain. Step 2: Carefully separate the brain from the space between the two eye-antennal discs using dissecting needles. Step 3: Use dissecting needles to cut off the eye-antennal discs from the mouth hooks. Step 4: Transfer the isolated eye-antennal discs into a fresh droplet of culture medium using a 100 μL pipette. Created in BioRender. Sui, L. (2025) https://BioRender.com/3cnp72p.

**Figure 2:**
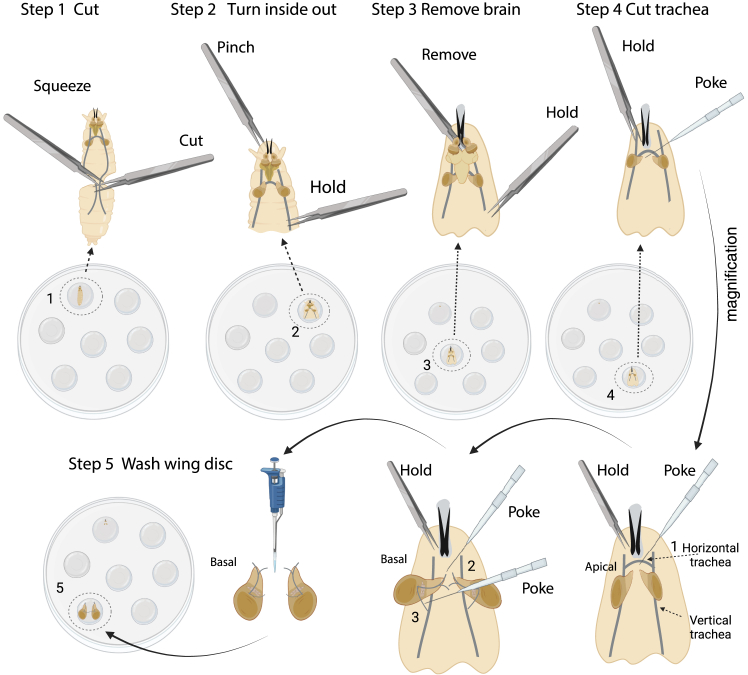
Dissection of wing discs for *ex vivo* culture Step-by-step procedure for isolating wing discs from third instar larvae. Step 1. Use two pairs of forceps to cut larva in half to expose the internal tissues. Step 2. Invert the larval body by holding the mouth hooks with one pair of forceps and gently pushing the larval body wall with the second pair of forceps towards the mouth hooks. Step 3. Carefully remove the gut, fat body and brain to expose the wing discs. Step 4. Cut tracheae. First, use a dissecting needle to cut the horizontal trachea. Then, gently poke off the trachea connecting the larval body wall with the notum tip of the wing disc. Finally, severe the tracheal connection between adult muscle precursor cells of the wing disc and the main vertical trachea. Step 5. Transfer the isolated wing discs into a fresh droplet of culture medium using a 100 μL pipette. Created in BioRender. Sui, L. (2025) https://BioRender.com/ah3k28z.

2. Imaginal disc dissection.
  a. For eye-antennal disc dissection
    i. Move one larva into a new droplet of culture medium.
    ii. Gently hold the larval body at a point approximately one-third of the distance from the anterior end using one pair of forceps, grab the mouth hooks with a second pair of forceps and pull the mouth hooks out. (Figure 1-Step 1). The mouth hooks, the attached pair of eye-antennal discs and the larval brain should now be removed from the larval body.
    iii. Carefully remove the fat body and gut from the eye-antennal discs and the attached brain.
    iv. Transfer the eye-antennal discs and the attached brain into a new droplet of culture medium.
    v. Hold the mouth hooks by one pair of forceps. Use dissecting needles to separate the eye-antennal discs from the brain (Figure 1-Step 2).
    vi. Cut off the eye-antennal discs from the mouth hooks using dissecting needles (Figure 1-Step 3).
    vii. Transfer the eye-antennal discs into a fresh droplet of culture medium using a 100 μL pipette (Figure 1-Step 4).
  b. For wing disc dissection
    i. Use two forceps to cut larvae in half, preserving the anterior which contains the mouth hooks (Figure 2-Step 1).
    ii. Hold the mouth hooks with one pair of forceps and gently push the larval body wall with the second forceps over the mouth hooks, turning the larva inside-out (Figure 2-Step 2).
    iii. Carefully remove brain, fat body, gut and other imaginal discs (Figure 2-Step 3).
    iv. Gently break the horizontal trachea between the two main vertical tracheae using a dissecting needle (Figure 2-Step 4.1).
    v. Turn larval body to see the basal side of the wing disc. Gently break the trachea connecting the notum tip of the wing discs and the body wall using a dissecting needle (Figure 2-Step 4.2).
    vi. Gently break the trachea connecting the adult muscle precursor cells of the wing disc with the main vertical trachea (Figure 2-Step 4.3).
    vii. Transfer the wing discs into a fresh droplet of culture medium using a 100 μL pipette (Figure 2-Step 5).

***Note:*** Do not pull the trachea away from the wing discs using forceps.

**CRITICAL:** Frequently transfer the wing discs to fresh droplets of culture medium during dissection.

**Figure 3:**
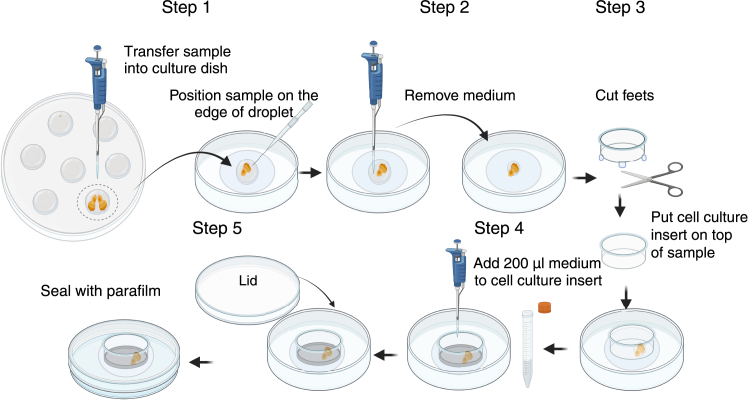
Mounting of imaginal discs using cell culture insert Step 1. Using a pipette, transfer imaginal discs onto glass-bottom microwell dish. Gently position them on the edge of a droplet of culture medium using a dissecting needle. Step 2. Remove excess medium from the droplet using a pipette to flatten the sample. Step 3. Cut the feet of the cell culture insert and place it on the top of samples. Step 4. Quickly add 200 μL culture medium to cell culture insert. Step 5. Place the lid onto the dish and seal with parafilm. The imaginal discs are now ready for imaging. Created in BioRender. Sui, L. (2025) https://BioRender.com/kzzc7y3.

**Figure 4:**
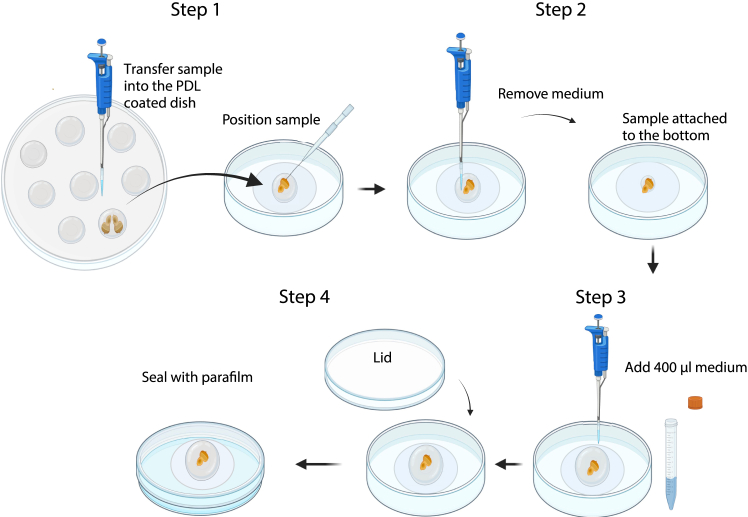
Mounting imaginal discs on Poly-D-Lysine coated dishes Step 1. Using a pipette, transfer imaginal discs in 50 μL of culture medium onto a Poly-D-Lysine coated dish. Use a dissecting needle to gently position the imaginal discs to the center of the droplet, with either the apical or basal side facing the bottom of the dish as appropriate for imaging. Step 2. Carefully remove excess medium from the droplet using a pipette to flatten the imaginal discs and to facilitate their attachment to the bottom of the dish. Step 3. Quickly add 400 μL of culture medium on top of the attached imaginal discs. Step 4. Place the lid onto the dish and seal it with parafilm. The imaginal discs are now ready for imaging. Created in BioRender. Sui, L. (2025) https://BioRender.com/gdd8u4w.

3. Mounting imaginal discs.
  ***Note:*** We detail in the following two procedures to mount imaginal discs.
  a. For mounting using a cell culture insert (modified based on Zartman et al., 2013^11^).
    i. Transfer the imaginal discs within 20 μL of culture medium onto a glass-bottom microwell dish using a 100 μL pipette. Flatten the droplet using the pipette (Figure 3-Step 1).
    ii. Move imaginal discs to the edge of the droplet by pulling on the trachea of the notum tip (wing disc) or by pulling on the optic stalk (eye-antennal disc) using a dissecting needle. Turn imaginal disc apical up or basal up as appropriate for imaging (Figure 3-Step 1).
    iii. Remove the remaining culture medium from the dish using a 100 μL pipette to flatten the imaginal disc (Figure 3-Step 2).
      ***Note:*** Be careful to ensure that the imaginal disc remains surrounded by some residual culture medium to prevent it from drying out.
    iv. Cut off the feet of the cell culture insert. Keep around 40 μm remaining feet as a spacer. Place the cell culture insert on top of the imaginal discs (Figure 3-Step 3).
    v. Quickly fill 200 μL culture medium into the cell culture insert (Figure 3-Step 4).
      **CRITICAL:** Ensure that the refilled culture medium completely surrounds the imaginal disc without any air gaps. If air bubbles are present at the bottom, add more culture medium from the side to eliminate them.
    vi. Place the lid onto the glass-bottom microwell dish and seal with parafilm (Figure 3-Step 5).
  b. For mounting imaginal disc on Poly-D-Lysine coated dishes
    i. Transfer imaginal discs within 50 μL of culture medium onto a Poly-D-Lysine coated dish using a pipette (Figure 4-Step 1).
      ***Note:*** Using less medium may cause the imaginal discs to attach to the Poly-D-Lysine coated dish before they can be properly positioned.
    ii. Gently flow the medium around the imaginal discs using a dissecting needle to orient the imaginal discs with the apical side up or the basal side up as appropriate for imaging (Figure 4-Step 1).
    iii. Remove the remaining culture medium from the dish using a pipette (Figure 4-Step 2).
    iv. Quickly add 400 μL culture medium into the dish (Figure 4-Step 3).
    v. Place the lid onto the dish and seal with parafilm (Figure 4-Step 4). Troubleshooting 5.
      **CRITICAL:** Mounting imaginal discs using a cell culture insert allows for the observation of cell behaviors both at the apical and basal side of the tissue. However, the imaginal discs can more easily tilt or float away from the bottom of the dish. By contrast, imaginal discs mounted on Poly-D-Lysine coated dishes adhere more easily and stably to the bottom of the dish, thereby facilitating imaging. However, this latter procedure cannot be used to observe dynamic processes at the basal side of cells.
4. Live imaging using a confocal microscope. Troubleshooting 6 and 7.
  To observe cell and tissue shape changes with cellular resolution for up to 15 to 20 h, acquire images or image stacks fast and with low laser power to minimize phototoxicity. We use Leica Sp5 and Sp8 inverted confocal laser-scanning microscopes with a 40x/1.25 oil-immersion objective or a 60x/1.25 water objective within a temperature controlled imaging chamber.
  a. Place the 35 mm dish containing the imaginal discs on the stage of an inverted confocal microscope.
  b. Select appropriate lasers (e.g.: 488 nm for GFP excitation and 565 nm for RFP excitation).
  c. Use low laser power (we use < 5%) to minimize phototoxicity.
  d. Set format size to 1024∗700 pixels to include region of interest of imaginal disc.
    ***Note:*** Decreasing the pixel number along the y-axis will shorten the scanning time while maintaining image resolution.
  e. Adjust zoom factor to reach an image resolution between 3.5 pixel/μm - 5.5 pixel/μm.
  f. Set range of z-stack acquisition to image the entire depth of imaginal disc. We usually use 20 to 50 image slices with 1.5 μm spacing.
  g. Set 5 or 10 min interval time for total period of 20 h for long term imaging.
    ***Note:*** We typically use acquisition times for a single z-stack of 1 to 1.5 min. If multiple samples are imaged together by tile scan, use interval times between acquisition of a given sample of 5 to 10 min, as appropriate. Prolong the interval time or decrease laser power in case phototoxic effects are observed.
    ***Note:*** For observing protein dynamics of the fluorescent markers, set format size to 1024∗250 pixels to shorten the image acquisition time. We regularly image with an acquisition time of 20 s for an entire z-stack, at interval times of 30 s or 60 s for a total time period of 3 h.

### Denoising and restoration of axial resolution of images by Fiji plugin CSBDeep

**Timing: 24 h**

Volumetric time-lapses are acquired using low laser intensity and few z-slices to minimize phototoxicity. However, this results in raw images that exhibit significant noise and low z-axis resolution. Denoising and restoration of raw images can lower noise and improve z-axis resolution. We outline the use of a machine-learning-based image restoration algorithm termed Content-aware image restoration (CARE^12^). See also the Fiji plugin-CSBDeep.^10^^,^^12^

**Figure 5:**
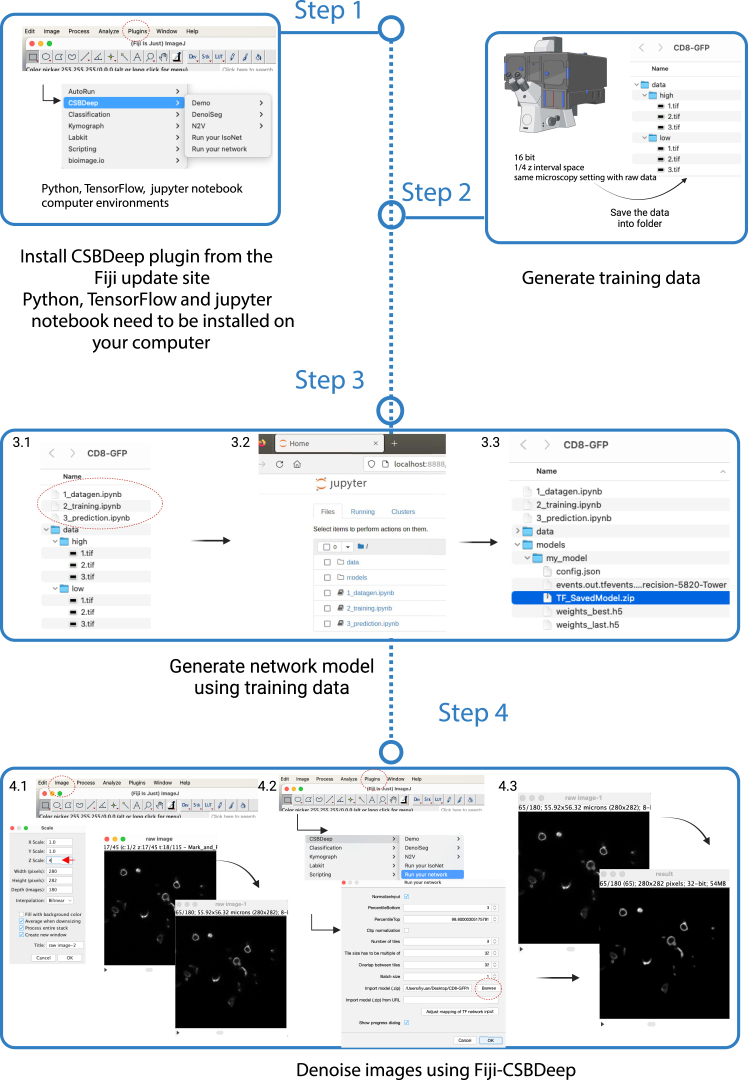
The procedure of denoising and restoration of axial resolution of images by Fiji plugin-CSBDeep Step 1. Install CSBDeep plugin from the Fiji update site. Step 2. Generate training data. Step 3. Generate network model using training data. Step 4. Denoise images using Fiji-CSBDeep. Created in BioRender. Sui, L. (2025) https://BioRender.com/sqqv7yh.

5. Generate training data (Figure 5-Steps 1 and 2).
  a. Prepare a dish with cultured imaginal discs that express the same fluorescence markers as acquired in the raw images.
  b. Set up acquisition of 16 bit-images.
  c. Set up the same microscope settings as for acquisition of the raw images.
  d. Set up to acquire two sets of training data at the same time using two channels: Channel 1. Low laser power (as for acquisition of the raw images). Channel 2. High laser power.
    ***Note:*** Image quality is significantly enhanced in the high laser power channel, but avoid capturing overexposed images. Ensure that the microscope settings for both channels are identical except for the laser power: one should be set to low, and the other to high.
  e. Set up distance between z-slices of the image stack to one quarter of the z-stack interval of the raw images (i.e. if the z-stack interval of the raw images is 1.5 μm, then use an interval of 0.375 μm for acquiring the training data). This procedure increases z-axial resolution in the image data.
  f. Set up “frame scan” for scanning these two channels. Images in the frame must include a black background surrounding the tissue.
  g. Acquire entire image stack.
    ***Note:*** Ensure that both channels scan simultaneously, as the live tissue may dynamically change shape and position during imaging.
    ***Note:*** We usually acquire 5 different training data (i.e. image stacks) for each marker. More training data may improve the training model.
  h. Create “data” folder with subfolder “low” and “high” for the training data (here: “CD8-GFP” folder).
  i. Split the two channels of the training data. Save image stack acquired with low laser power into “low” folder and save image stack acquired with high laser power into “high” folder (Figure 5-Step 2).
6. Generate network model using training data (Figure 5-Step 3).
  ***Note:*** Training data can be acquired from any specific organism, tissue, fluorescent marker, and microscope setting.
  ***Note:*** Denoising and restoration of images with different fluorescent markers or microscopy settings requires the use of a corresponding training model.
  a. Find the workflow from the unsampling 3d folder (https://github.com/CSBDeep/CSBDeep/tree/main/examples).
  b. Copy three notebooks into “CD8-GFP” folder (Figure 5-Step 3.1).
    1_datagen.ipynb
    2_training.ipynb
    3_prediction.ipynb
  c. Open “CD8-GFP” in a terminal.
  d. In the terminal (still in the CD8-GFP folder), start the jupyter server by typing “jupyter notebook”.
  e. A browser window appears and starts to run “1_datagen.ipynb” (Figure 5-Step 3.2).
  f. Run 1_datagen.ipynb by Shift-enter through the jupyter notebook (change the data folder name).
  g. Run 2_training.ipynb by Shift-enter through the jupyter notebook (training might take ∼1 h).
  h. The training model will get exported to “models/my_model/TF_SavedModel.zip” after finishing to run 2_training.ipynb (Figure 5-Step 3.3).
7. Denoise images on Fiji-CSBDeep (Figure 5-Step 4).
  a. Load your 4D image stacks on Fiji.
  b. Go Fiji- images- Scale-Z-scale-put a factor 4 (Figure 5-Step 4.1).
  c. Go Fiji plugin-CSBDeep-> Run your network (Figure 5-Step 4.2).
  d. “Import model”->select “TF_SavedModel.zip” in your models folder (e.g. CD8-GFP/models/my_model/ TF_SavedModel.zip) (Figure 5-Step 4.2).
  e. Denoised images get exported from Fiji as a “result” image stack (Figure 5-Step 4.3).
  f. Adjust the brightness of “result” images by clicking Reset.
  g. Convert 32 bit “result” into 8 bit.

***Note:*** The denoised images have a ∼4-fold increased resolution along the z-axis compared with the raw images.

## Expected outcomes

A successful live imaging experiment will allow to observe and analyze early third instar imaginal discs for 14–16 hours or late third instar imaginal discs for 18–20 h. During the entire period of culturing imaginal discs, a considerable number of cell divisions should be observed. However, it is typical to see a gradual decrease in the number of dividing cells after 2–5 h of cultivating late third instar wing discs. Moreover, for early third instar wing discs, the formation of epithelial folds should be observed.^13^ For eye-antennal discs, the movement of the morphogenetic furrow and the formation of photoreceptor clusters should be detectable.^1^

**Figure 6:**
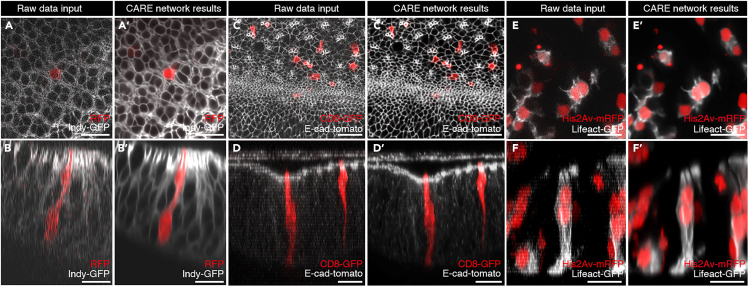
Denoising and restoration of axial resolution of images with Fiji plugin-CSBDeep (A–B′) x-y (A and A′) and x-z (B and B′) views of input raw data (A and B) and restorations (A′ and B′) of eye-antennal disc expressing Indy-GFP to mark cell membranes (gray) and carrying clones of cells expressing RFP (red). (C–D′) x-y (C and C′) and x-z (D and D′) views of 3D projections of input raw data (C and D) and restorations (C′ and D′) of eye-antennal disc expressing E-cadherin-tomato to mark adherens junctions (gray) and carrying clones of cells expressing CD8-GFP (red). (E–F′) x-y (E and E′) and x-z (F and F′) views of 3D projections of input raw data (E and F) and restorations (E′ and F′) of eye-antennal disc carrying clones of cells expressing His2Av-mRFP to mark nuclei (red) and Lifeact-GFP to mark F-actin (gray). Scale bars: 10 μm.

High-quality denoised images should be obtained after image restoration using Fiji-CSBDeep, even when the raw images display considerable noise. Moreover, the z-axial resolution of the image stack should be increased. Denoising and axial image restoration should improve observation and image interpretation, and should facilitate downstream image processing, including segmentations and quantification of cellular and tissue morphometric parameters. Fiji-CSBDeep enables high-quality images with limited laser power, and thereby helps to minimize phototoxicity allowing long-term imaging (Figure 6).

## Limitations

It is challenging to obtain single-cell clones in wing discs from early third instar larvae. The clones typically consist of 2 or 4 cells. GFP synthesis takes ∼7 hours following heat shock-induced Flp-mediated recombination, and a strong GFP signal can be detected 12 hours after recombination. By this time, the cells have already undergone one or two round of cell division, resulting in clones that consistently contain 2 to 4 cells in the early third instar wing discs.

Although the imaginal discs can develop for approximately 18–20 h *ex vivo* in culture medium, their development might be slower in culture medium compared with the *in vivo* situation inside the larva.

Fluorescent markers displaying a very weak signal, even when using high laser power, cannot yield good training data. Consequently, denoising and image restoration using Fiji-CSBDeep will also not produce good output results.

## Troubleshooting

### Problem 1

Cultured early third instar imaginal discs contain dead cells, and height and volume of cells decrease with time.

### Potential solution

Early third instar imaginal discs are sensitive to the osmolarity of the culture medium. A too high osmolarity of the culture medium may cause cell death and tissue shrinkage. Add an additional 1% sterile water to the culture medium.

### Problem 2

Cultured early third instar imaginal discs show abnormal tissue bending and cell death.

### Potential solution

Early third instar imaginal discs are sensitive to the concentration of 20-Hydroxyecdysone. A too high concentration of 20-Hydroxyecdysone in the medium causes cell death and tissue bending in early third instar imaginal discs. Therefore, the final concentration of 20-Hydroxyecdysone should be 20 nM for culturing early third instar imaginal discs. A lower concentration may not support proper cell proliferation. Moreover, during dissection, the accidental scratching with a dissecting needle of the ‘peripodial membrane’, which lies on top of the ‘disc proper’, can initiate wound healing. Wound healing exerts contractile force that can lead to abnormal tissue bending. In such cases, it is best to discard the affected imaginal disc and carefully dissect a new one.

### Problem 3

Cultured early third instar imaginal discs do not show dynamic cell behaviors including cell division.

### Potential solution

The culture medium may be contaminated. Check the transparency of the culture medium and inspect for floccules. If medium contamination is suspected, prepare a fresh culture medium and ensure that it has been sterilized through filtration. Ensure that forceps and dissecting needles are sterilized and larvae are sufficiently washed. Moreover, during dissection, frequently transfer the tissue to the fresh droplets of culture medium.

### Problem 4

Imaginal discs attach to the bottom of the Poly-D-Lysine coated dish before they can be properly positioned.

### Potential solution

Ensure that imaginal discs are transferred to the culture dish by using a large volume of culture medium (approximately 50 μL). Once in the dish, position the imaginal disc properly (apical or basal side facing the bottom of the dish as appropriate for imaging) using a dissecting needle.

### Problem 5

Imaginal discs do not attach to the bottom of the Poly-D-Lysine coated dish, and ‘flow up’ when culture medium is added.

### Potential solution

Ensure that the entire surface of the dish is fully covered by Poly-D-Lysine during coating. Moreover, allow the Poly-D-Lysine coated dishes to completely dry before use. Do not reuse Poly-D-Lysine coated dishes. If the mounting process fails, start over using a new Poly-D-Lysine coated dish.

### Problem 6

Fluorophore signal declines over time during live imaging.

### Potential solution

The decline of fluorophore signal may be caused by photobleaching. To limit the laser exposure to the sample, lower the laser power, increase the time interval between imaging frames, reduce the range of the z-stack or increase the distance between z-slices.

### Problem 7

The sample drifts during live imaging.

### Potential solution

Ensure that the sample is stably mounted at the bottom of the dish. If not, it is best to repeat the mounting using a freshly dissected imaginal disc.

## Resource availability

### Lead contact

Further information and requests for resources and reagents should be directed to and will be fulfilled by the lead contact, Christian Dahmann (christian.dahmann@tu-dresden.de).

### Technical contact

Technical questions on executing this protocol should be directed to and will be answered by the technical contact, Liyuan Sui (liyuan.sui@tu-dresden.de).

### Materials availability

This study did not generate new unique reagents.

### Data and code availability

This study did not generate original code.

## Acknowledgments

We thank Bloomington Drosophila Stock Center for fly stocks and the light microscopy facility of the Biotechnology Center at TU Dresden for technical assistance. The graphical abstract was created in BioRender. Sui, L. (2025) https://BioRender.com/ax8k52a.

C.D. was supported by the 10.13039/501100001659Deutsche Forschungsgemeinschaft under Germany’s Excellence Strategy—EXC-2068-390729961—Cluster of Excellence Physics of Life of TU Dresden.

## Author contributions

L.S. optimized the protocol for dissecting, mounting, and imaging of imaginal discs. L.S. performed the experiments, analyzed the data, and wrote the first draft of the manuscript. C.D. and L.S. wrote the final manuscript.

## Declaration of interests

The authors declare no competing interests.
